# Supplementary material for: Impact of Surface Passivation on the Efficiency and High-Speed Modulation of III–V GaAs/AlGaAs Nanopillar Array LEDs
Source: ACS Photonics. 2025 Nov 5;12(11):6269–77. doi: 10.1021/acsphotonics.5c01751 (PMC12776361; doi:10.1021/acsphotonics.5c01751)
Supplement: Supplementary file 1 [file ph5c01751_si_001.pdf]

# Supporting Information for “Impact of Surface Passivation on the Efficiency and High-speed Modulation of III–V GaAs/AlGaAs Nanopillar Array LEDs”

*Bejoys Jacob,[1,2]* João Azevedo,[1,3] João Lourenço,[1,2] Filipe Camarneiro,[1] Jana B. Nieder,[1] Bruno Romeira[1]***

*[1]INL – International Iberian Nanotechnology Laboratory, Av. Mestre José Veiga s/n, 4715-330, Braga, Portugal*

*[2]Centro-Ciências and LIP - Laboratório de Instrumentação e Física Experimental de Partículas, Departamento de Física, Faculdade de Ciências, Universidade de Lisboa, 1749-016 Lisboa, Portugal*

*[3]Departamento de Física, Universidade do Minho, Campus de Gualtar, 4710-057, Braga, Portugal*

**bejoys.jacob@inl.int, ** bruno.romeira@inl.int*

## S1. Epilayer design

The III-V epilayer semiconductor compound material stack for the *p-i-n*-type nanoLED (Be dopant for *p*-type layers and Si dopant for *n*-type layers) was grown by metalorganic chemical vapour deposition (MOCVD) on a semi-insulating (SI) GaAs substrate and is shown in Table S.1 (the active emitting layer is highlighted).

Table S1: Epilayer stack of the *p-i-n*-type nanopillar array LED devices.

| Layer # | Thickness (nm) | Semiconductor | Doping ($cm^{-3}$) | Description |
|---|---|---|---|---|
| 23 | 150 | GaAs | $5 \times 10^{18}$ : Be | Collector – $p^{++}$ |
| 22 | 20 | GaAs | $5 \times 10^{17}$: Be | $p^{+}$ |
| 21 | 20 | $Al_{0.1}Ga_{0.9}As$ | $5 \times 10^{17}$ : Be | Confinement layer |
| 20 | 20 | $Al_{0.2}Ga_{0.8}As$ | $5 \times 10^{17}$ : Be | Confinement layer |
| 19 | 100 | $Al_{0.2}Ga_{0.8}As$ | $5 \times 10^{16}$ : Be | Confinement layer |
| **18** | **280** | **GaAs** | **n.i.d.** | **Active emitting layer** |
| 17 | 100 | $Al_{0.2}Ga_{0.8}As$ | $2 \times 10^{16}$ : Si | Confinement layer |
| 16 | 20 | $Al_{0.2}Ga_{0.8}As$ | $2 \times 10^{17}$ : Si | Confinement layer |
| 15 | 20 | $Al_{0.1}Ga_{0.9}As$ | $2 \times 10^{17}$: Si | Confinement layer |
| 14 | 20 | GaAs | $2 \times 10^{17}$ : Si | $n^{+}$ |
| 13 | 100 | GaAs | $3 \times 10^{18}$ : Si | $n^{++}$ |
| 12 | 10 | GaAs | $1 \times 10^{17}$: Si | $n^{+}$ |
| 11 | 10 | GaAs | $1 \times 10^{16}$ : Si | $n^{-}$ |
| 10 | 5 | GaAs | n.i.d. | Spacer |
| 9 | 1.7 | AlAs | n.i.d. | Barrier |
| 8 | 5.5 | GaAs | n.i.d. | Quantum well |
| 7 | 1.7 | AlAs | n.i.d. | Barrier |
| 6 | 5 | GaAs | n.i.d. | Spacer |
| 5 | 10 | GaAs | $1 \times 10^{16}$ : Si | $n^{-}$ |
| 4 | 10 | GaAs | $1 \times 10^{17}$ : Si | $n^{+}$ |
| 3 | 200 | GaAs | $3 \times 10^{18}$ : Si | Emitter – $n^{++}$ |
| 2 | 100 | $Al_{0.75}Ga_{0.25}As$ | n.i.d. | Sacrificial layer |
| 1 | 130 | GaAs | n.i.d. | Buffer |
| | | GaAs | SI | Substrate |

## S2. Fabrication

Figure S1 illustrates the schematic diagram of the fabrication procedure for the *p-i-n* bipolar nanopillar array LEDs (nanoLEDs). Square samples (2 cm × 2 cm) were initially cleaned in an ultrasonic bath with acetone for 5 min, followed by isopropyl alcohol (IPA) for 5 min, and then deionized (DI) water for 3 min (Figure S1 (i)). After additional surface cleaning by rapid oxygen plasma (13 min) and deoxidation in ammonium hydroxide solution (1:10 in volume), samples were coated with a layer of $SiO_2$ (~200 nm) using a plasma enhanced chemical vapour deposition (PECVD) tool (SPTS, MPX CVD Module). Before photoresist coating, the samples were coated with hexamethyldisilazane (HMDS) vapour (YES, 310TA) to improve adhesion of the 500 nm thick negative e-beam resist (ARN7520.18). The samples were then subjected to e-beam lithography (Vistec, EBPG5200) to define the size and shape of the nanopillars. Using a reactive ion etching (RIE) tool (SPTS, APS Module) this pattern was later transferred to the $SiO_2$ layer to obtain the hard mask. The samples were dry etched using ICP-RIE (SPTS, ICP Module) until a height of 960 nm (i.e. until reaching the $n^+$- doped GaAs layer in the bottom layer) (Figure S1 (ii)). The $SiO_2$ hard mask was then removed from the top of the micropillars using HF vapour treatment (SPTS, Primaxx uEtch). The samples were then deoxidized with ammonium hydroxide (1:10) solution for ~4 min, followed by an ammonium sulphide (1:10) solution (for ~5 min at 65 °C) to chemically passivate its surface, and then immediately coated with a thin dielectric layer of $Si_xN_y$ (~100 nm) using low-frequency (380 kHz) PECVD (SPTS, MPX CVD Module) deposition(Figure S1 (iii)) [1]. We note in this process, no rinsing with water was used to preserve the sulphide layer formed in the GaAs surface. The sample was cleaned only using $N_2$ gun. After the treatment the sample was immediately transported to the PECVD deposition load lock chamber and the sample is pumped to vacuum conditions for PECVD deposition of low frequency $Si_xN_y$ layer. In all our tests, the typically time for air exposure before the dielectric coating is less than 5 min.

To open a via on top of the nanopillar array LED we have used a planarized etch back step using ICP-RIE dry etching technique (Figure S1 (iv-vi)). Firstly, the nanoLED samples, which are passivated with the dielectric layer, were coated by a thick (~3 µm) photoresist (AZ1505) (Figure S1 (iv)). Then, using controlled oxygen plasma reactive ion etching (SPTS, APS Module), a uniform etch of the thick photoresist was realized until reaching the top of the nanopillars (Figure S1 (v)). The etching rate was monitored using a contact profilometer and optical microscope inspection until the top portion of the dielectric-coated pillar was visible, while the rest of the pillars were submerged in the thick photoresist. To open the via for the top contact of the nanopillars, we etched the 100 nm-thick dielectric layer on top of the nanopillars by RIE (SPTS, APS Module) (Figure S1 (vi)). The photoresist leftover was washed away by acetone, IPA, and DI water. The samples were then cleaned by oxygen plasma leaving us with a sample containing nanopillars with an open top via (Figure S1 (vii)).

To define the bottom and top contacts (as well as the bottom via opening for the bottom contacts through the dielectric layer), we employed optical lithography by direct laser writing (DLW) method (Heidelberg instruments, DWL2000). We have used a physical vapor deposition tool (Kenosistec, UHV multi-target confocal sputtering) to deposit the top and bottom metal contacts on the nanopillar LEDs (Figure S1 (ix-x)). Before the metal deposition, the sample was treated with ammonium hydroxide solution (1:10) for 4 min and ammonium sulphide solution (1:10) at 45 °C for 5 min to remove any additional oxide layers that could contaminate the semiconductor contact surface. For the *n*-type bottom contact we used the metal alloy composition Ge(20 nm)/Ni(10 nm)/Au(150 nm), whereas for the *p*-type top contact, we used Pt(10 nm)/Ti(10 nm)/Pt (10 nm)/Au (100 nm). The top contacts were deposited at an angle (~45° relative to the vertical direction) to enable light extraction from the sidewalls of the nanopillars (Figure 2 in the main article). After each metal sputtering for top and bottom contact, the samples were kept in acetone for lift-off of the photoresist. Lastly, to improve the

contact series resistance of fabricated nanoLED, the samples were thermally annealed at 400 °C for 30 s in an $N_2$ atmosphere.

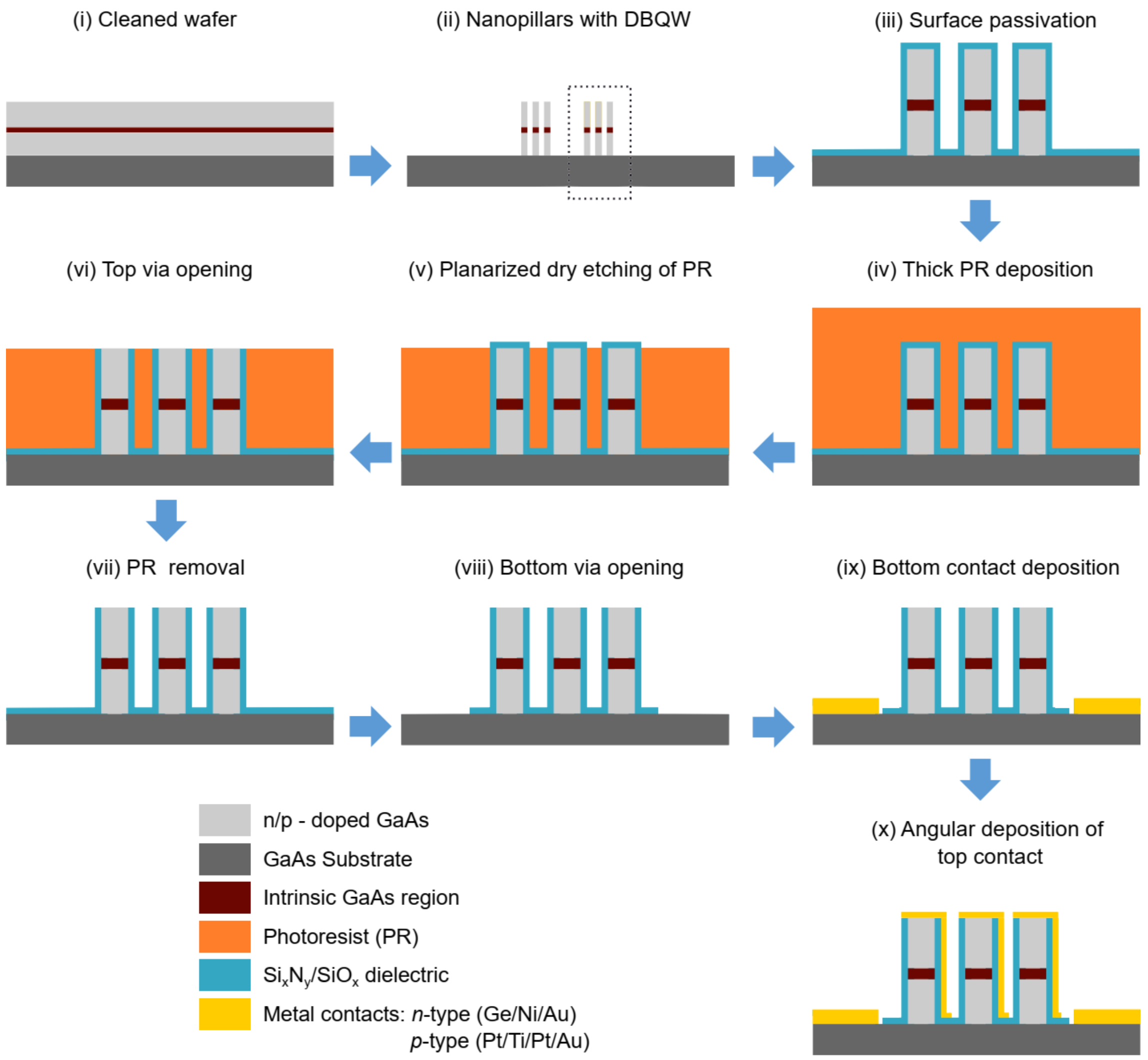


***Figure S1:*** *Fabrication process flow of nanopillar array LEDs.*

## S3. Static characteristics: Electroluminescence and L-I-V characteristics

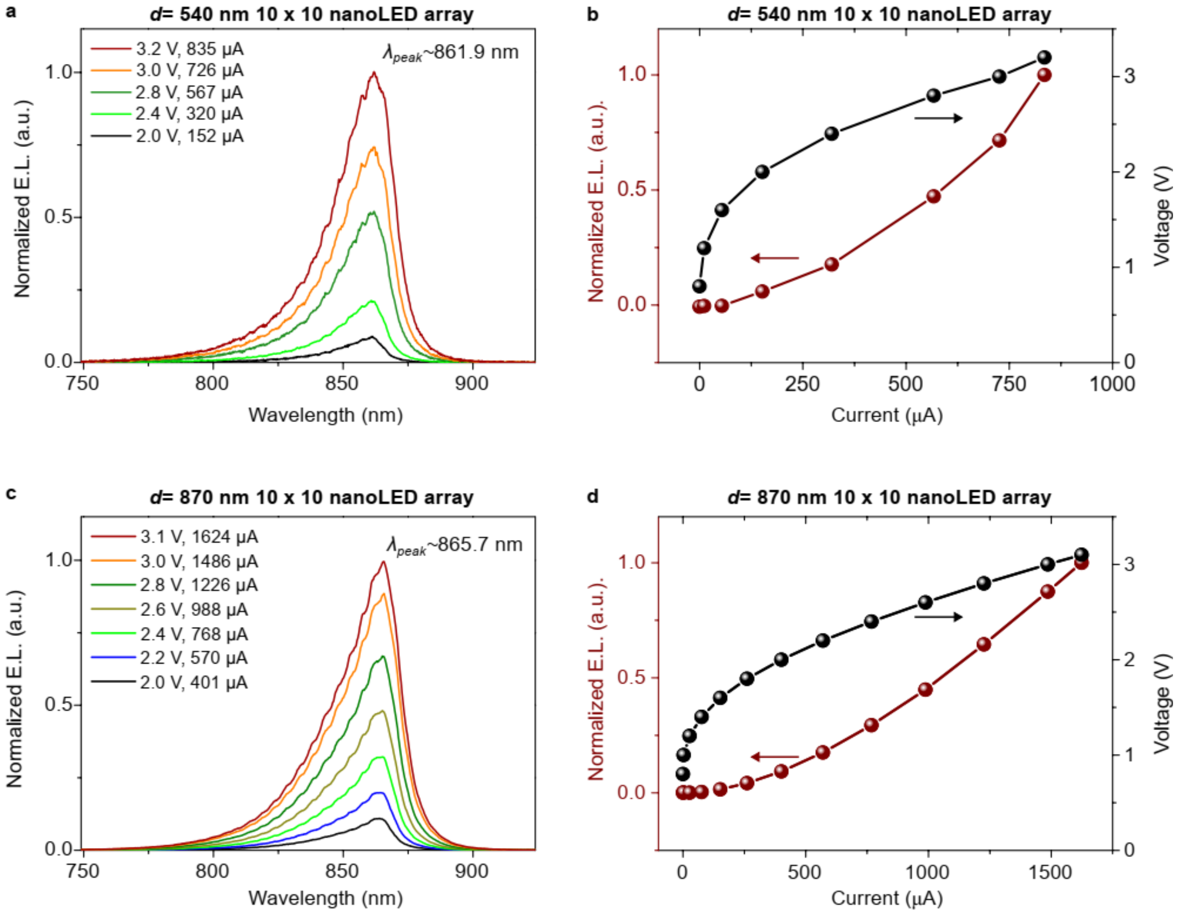


***Figure S2:*** *Static characteristics of nanoLEDs.* ***(a)*** *Electroluminescence spectra under different bias conditions for d=540 nm 10x10 nanopillar array LEDs.* ***(b)*** *L-I-V characteristics of d=540 nm 10x10 nanopillar array LEDs.* ***(c)*** *Electroluminescence spectra under different bias conditions for d=870 nm 10x10 nanopillar array LEDs.* ***(d)*** *L-I-V characteristics of d=870 nm 10x10 nanopillar array LEDs.*

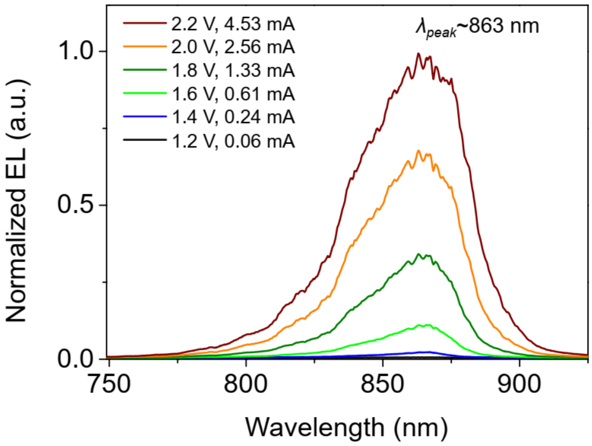


***Figure S3****: Electroluminescence spectra under different bias conditions for d=10 μm microLED device. The peak emission for the microscale LED ($\lambda_{peak}$~863 nm) matches that of the nanoscale LED devices considered in this work.*

We compared the electroluminescence (EL) spectra of microLED and nanoLED devices (Figure 4a and Figure S3) and extracted the full width at half maximum (FWHM). Figure S4 compares the FWHM as a function of the operating current density for the *d*=440 nm nanopillar array LED and the *d*=10 μm microLED. The FWHM increases monotonously with the current density for both nano- and microLED

cases, with the microLED showing typically larger FWHM values (>32 nm) than the nanoLED (<26 nm). Since both devices were fabricated from the same GaAs/AlGaAs epitaxial stack, the main factors affecting the spectral width of the GaAs bulk emitter are the carrier density and potentially the junction temperature. The nanoLED array example reported here was operated typically at lower current densities (≤1 kA/cm²) as compared to the microLED (>1 kA/cm²) to mitigate potential failures due to higher applied voltage (due to the larger series resistance of nanoLEDs). At these higher current densities, the microLED exhibits stronger band filling and increased self-heating, which broaden the emission spectrum (note that we use a bulk emitter), resulting in larger FWHM values (>32 nm).

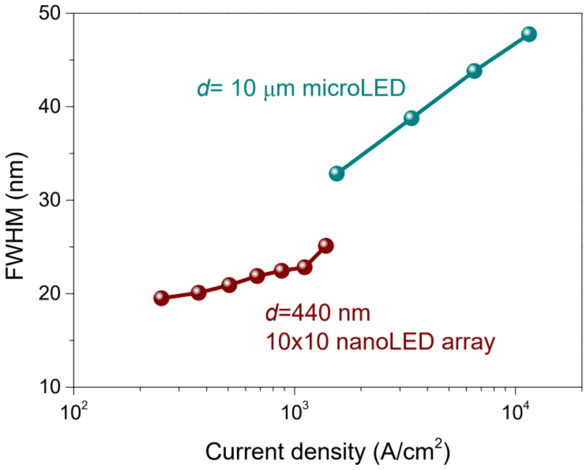


***Figure S4:*** *Full width at half maximum (FWHM) of the electroluminescence spectra as a function of current density for the d=440 nm nanopillar array LED and the d=10 µm microLED.*

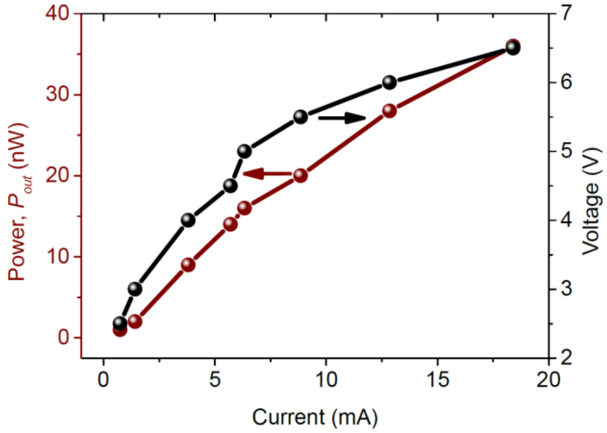


***Figure S5:*** *Example of a power-current-voltage (L-I-V) static characteristic for a 10x10 nanopillar array with pillar diameter with d=650 nm. The power measurement shown here was limited by the low numerical aperture (~0.2) of the lensed fiber used in our setup, the non-optimized collection angle of the fiber positioner fixed at ~15 ° from the normal axis, and additional optical losses from the metal-coated nanopillars.*

## S4. Dynamic response: high-speed modulation response

Table S2: Bandwidth of electrical components used for the electro-optical high-speed characterization.

| Component | Bandwidth (GHz) |
|---|---|
| Pulse generator (Active Technologies, model PG1072) | 2.3 (fall time $\tau_f$ <70ps) |
| Avalanche photodetector (MPD PSM series) | 4.5 (time resolution 35ps) |
| Bias-T (Minicircuits) | 0.001-4.2 |
| Coaxial cable (Minicircuits 141-2MSM+) | 18 |

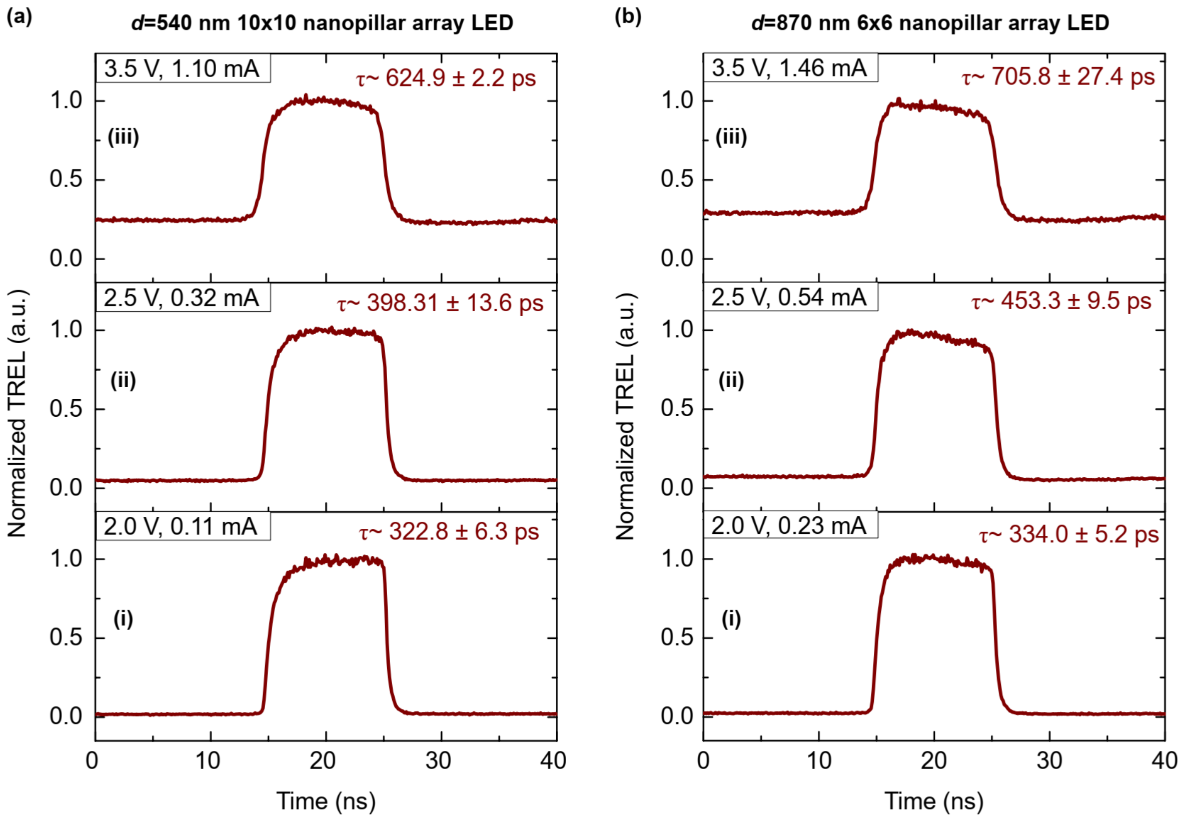


***Figure S6:*** *Time-resolved electroluminescence (EL) of the 10x10 nanopillar LED arrays, showing the decay lifetime (τ) as a function of the forward bias voltage ((i) 2 V, (ii) 2.5 V and (iii) 3.5 V).* ***(a)*** *Results obtained with the d=540 nm array LEDs (in a 10x10 array);* ***(b)*** *Results obtained with the d=870 nm array LEDs (in 6x6 array). Input electrical pulses of* $V_{pp}$*=1 V,* $t_{in}$*= 10 ns.*

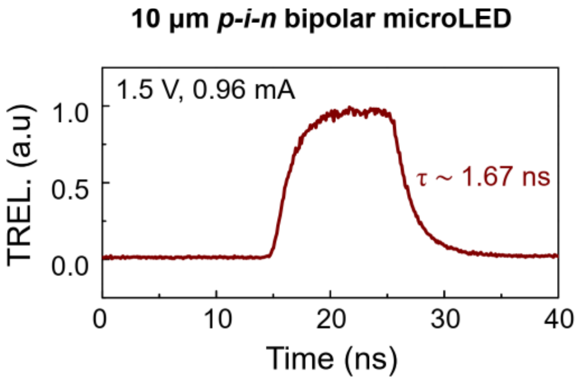


***Figure S7:*** *Time-resolved electroluminescence (EL) of a microLED (d=10 µm) showing the decay lifetime (τ~1.67 ns) for a low forward bias voltage 1.5 V, and input electrical pulses of* $V_{pp}$*=1 V,* $t_{in}$*= 10 ns. The microdevices are coated with* $SiO_2$ *by HF-PECVD instead of the* $Si_xN_y$ *films by LF-PECVD used for nanopillar LEDs.*

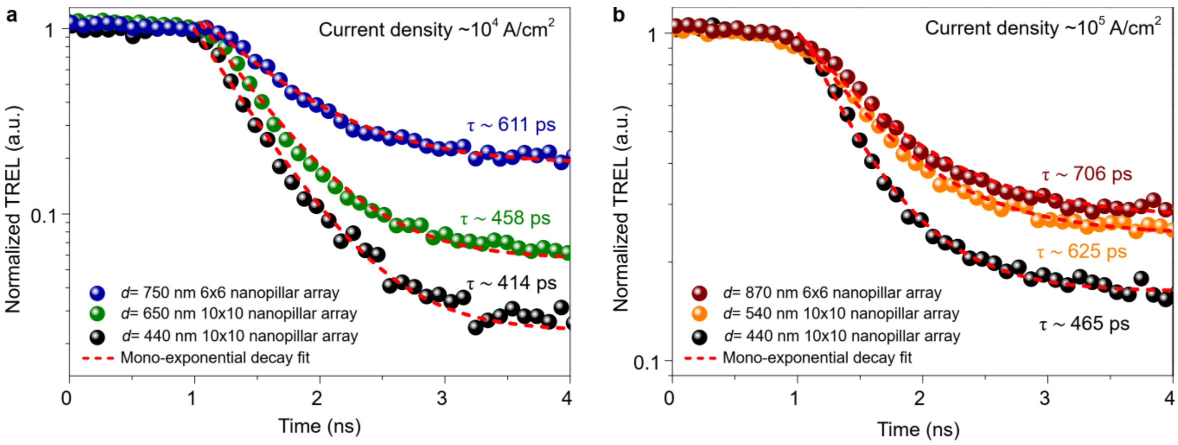


***Figure S8:*** *Time-resolved electroluminescence (EL) of the nanopillar LED arrays, showing the decay lifetime (τ) as a function of the size of the nanopillar array under similar current density conditions. (a) Decay traces for a current density of ~*$10^4$ *A/cm*$^2$ *for nanopillar array LEDs of size d=440nm (10×10 array), d=650 nm (10×10 array) and d=750 nm (6×6 array). (b) Decay traces for a current density ~*$10^5$ *A/cm*$^2$ *for nanopillar array LEDs of size d=440nm (10×10 array), d=540 nm (10×10 array) and d=870 nm (6×6 array).*

## S5 Coplanar waveguide (CPW) design of high-speed electrical contacts

For matching the impedance of our high-speed probes and to process high-speed signals, a 50 Ω coplanar waveguide (CPW) design for the electrical contacts of our nanoLED arrays was adopted. CPW is a type of electrical transmission line used for microwave-frequency signals. It consists of a central strip conductor surrounded by two ground planes, all on the same plane. The CPW structure provides advantages such as ease of fabrication, reduced dispersion, and good compatibility with integrated circuits. Finite ground CPW (FG-CPW) contacts (Figure S9) are CPWs with a finite ground size which are used as transmission lines for the devices discussed here. The key parameters of an FG-CPW include the width of the central signal line ($w$), the gap between the signal line and the ground planes ($s$), the width of the ground planes ($g$), the dielectric constant of the substrate ($\varepsilon_r$), and the thickness of the substrate ($h$). The width of the ground planes should be larger than the gap widths to ensure single-mode operation and is usually set to at least three times the gap spacing [2]. The impedance $Z_0$ and effective permittivity $\varepsilon_{eff}$ of FG-CPW on a finite substrate are given by equations [3]:

$$Z_0 = \frac{30\pi}{\sqrt{\varepsilon_{eff}}} \frac{K(k_1')}{K(k_1)} \quad \text{(E.1)}$$

$$\varepsilon_{eff} = 1 + \frac{\varepsilon_r - 1}{2}\frac{K(k')K(k_1')}{K(k)K(k_1)} \quad \text{(E.2)}$$

Here, $Z_0$ represents the characteristic impedance, $\varepsilon_{eff}$ denotes the effective permittivity, $\varepsilon_r$ is the relative permittivity of the substrate material, *K(k)* denotes the complete elliptic integral of the first kind for the value, *k*, *k'*, $k_1$, and $k_1'$ are parameters related to the dimensions and properties of the FG-CPW structure and the substrate.

$$k = \frac{w}{w+2s} \quad \text{(E.3)}$$

$$k_1 = \frac{\sinh\left(\frac{\pi w}{4h}\right)}{\sinh\left(\frac{\pi(w+2s)}{4h}\right)} \quad \text{(E.4)}$$

$$k' = \sqrt{1-k^2};\ k_1' = \sqrt{1-{k_1}^2} \quad \text{(E.5)}$$

The following dimensions were selected *w*=60 µm, *s*=40 µm, *g*=120 µm. Considering the relative dielectric constant of GaAs $\varepsilon_r$ =12.9 and thickness of substrate *h*≈675 µm, the estimated impedance is $Z_0$≈49.8 Ω, which closely matches the impedance of the electrical probes, coaxial cables and measurement systems used in the experiments (*Z*=50 Ω).

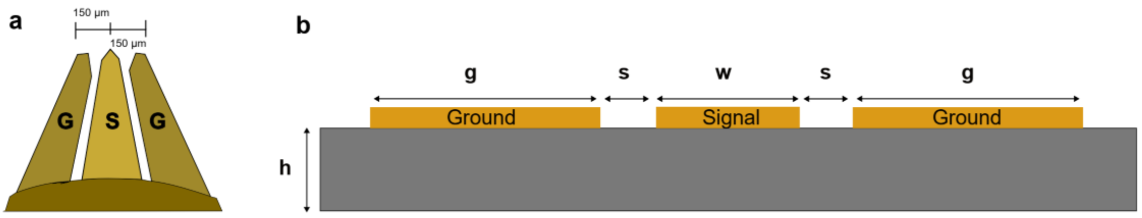


***Figure S9:*** *(a) Schematic of a G-S-G probe used for radio frequency (RF) characterization of optoelectronic devices. The pitch size of the probes is p=150 µm. (b) Cross-sectional image of FG-CPW contacts. The key defining parameters are the width of the central signal line (w=60 µm), the gap between the signal line and the ground planes (s) =40 µm, the width of the ground planes (g)=120 µm, the thickness of the substrate (h=675 µm) and the dielectric constant of the GaAs substrate ($\varepsilon_r$)=12.9.*

## S6 External quantum efficiency (EQE)

The EQE is given by:

$$\text{EQE} = \eta_c \text{IQE} \quad \text{(E.6)}$$

$\eta_c$ is the coupling coefficient and is determined by the numerical aperture (NA~0.2) of the lensed fiber used to collect the EL data (assuming an optimal collection angle from the sidewall pillars), ratio of extraction efficiency of the pillar ($\eta_{pillar}$) to the planar LED emission ($\eta_{bulk}$) and ratio of light coupled with metal coated pillars to uncoated ones ($\alpha$):

$$\eta_c = \frac{1}{4}\left(\frac{NA}{n}\right)^2 \left(\frac{\eta_{pillar}}{\eta_{bulk}}\right)\alpha \qquad \text{(E.7)}$$

where $n \approx 3.55$ is the refractive index of GaAs.

Following a methodology similar to previous work [4], $\eta_{pillar}$ was determined. By assuming the light extraction efficiency of the largest pillar diameter $d$=5 µm to be the same as $\eta_{bulk}\approx0.02$ and using the photoluminescence (PL) intensity per effective area plot for GaAs/AlAs nanopillars (see Figure S10), we can estimate the $\eta_{pillar}$ for smaller pillars. The PL experiments were performed in a confocal system (WITec Alpha 300R), using a 532 nm laser source operated at 150 µW, and the emission from the pillars was collected through an objective lens with NA=0.9. The collection path includes a UHTS300 spectrometer with a 600 lines/mm diffractive grating coupled to a Peltier cooled CCD detector. Since all pillars have a tapered shape, with a tilted angle $\theta_c$~21° relative to the vertical direction, their lateral emitting area is regarded as non-negligible for submicron cases ($d \leq 500$ nm). This allows for a realistic and reliable estimate of the actual PL intensity per area.

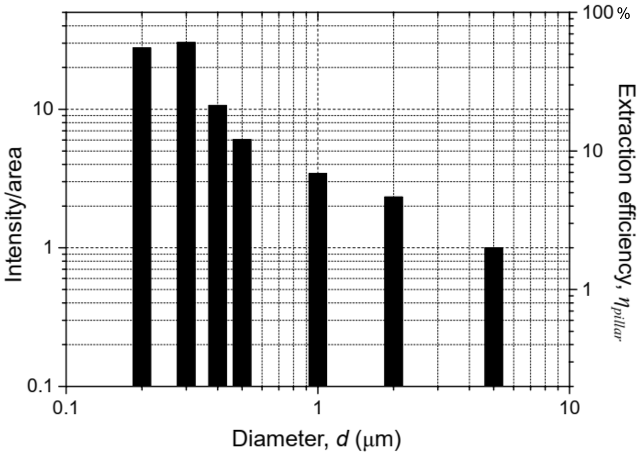


***Figure S10:*** *Integrated intensity per effective emitting area as a function of diameter for micro- and nanoscale pillars (d=200 nm to d=5 µm), and respective extraction efficiency (in percentage).*

To determine $\alpha$, ratio of the integrated PL measurements (see Figure S7), we considered the PL integrated intensity with and without metal coating for a $d_{top}$=500 nm pillar. For this analysis, an equivalent *p-i-n*-type nanoLED sample was tested but having different spacer layer thicknesses (top spacer: 15 nm; bottom spacer: 35 nm) and different well thickness (6.5 nm instead of 5.5 nm). This sample was processed through the same fabrication steps described in Section S.2 (see Figure S1), with a final etch depth of 1250 nm (slightly over-etched). The value $\alpha$~1/4.5 was obtained.

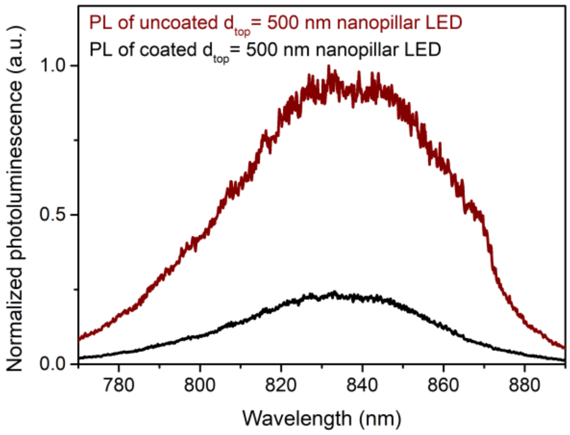


***Figure S11:*** *PL measurements of nanopillar LED with $d_{top}$ = 500 nm with and without top metal coating.*

Based on the above analysis of PL size-dependent measurements (Figure S10), the light out-coupling efficiency of uncoated pillars of size $d_{top}$~440 nm is estimated to be ~21 %. This values lies near to the results of our previous work, where an extraction efficiency in the 33%-57% range was shown in undoped GaAs/AlGaAs nanopillars [4]. This large extraction (as compared to the low extraction efficiency in planar microLEDs, ~2%), is mainly related to the strong reduction of the total internal reflection effect for nanopillars with sizes below 1 µm. However, in this work the electrically pumped nanoLEDs are coated with metal layers for the electrical contacts. Although a ~45° angle deposition of the metals was made to leave one of the sides of the nanopillars uncoated, PL measurements comparing uncoated and coated nanopillars reveal that the emission drops by a factor of ~4.5 (Figure S11), in which a fraction of this PL drop is related to the reduction of the illuminated area of the metal-coated nanopillar. As a result, considering also the limited NA aperture of the lensed fiber to couple light from the nanopillars, the estimated EQE (Equation E.6) for our devices is much lower than the reported IQE (EQE<$10^{-3}$). We note this estimated EQE is comparable with previously reported *p-i-n* III-V nanoscale light emitting devices, including *p-i-n* nanowire LED [5], *p-i-n* nanopillar metal-dielectric LED [6], *p-i-n* quantum dot PhC LED [7], which report EQEs ranging between $10^{-6}$ and $10^{-4}$. An estimate of EQE is shown in Figure S12. An EQE ranging from $0.25 \times 10^{-4}$ up to a maximum value of $8.4 \times 10^{-4}$ was observed in the highlighted pumping conditions ($N_d$=$10^{17}$ cm$^{-3}$ to $N_d$=$3\times10^{19}$ cm$^{-3}$).

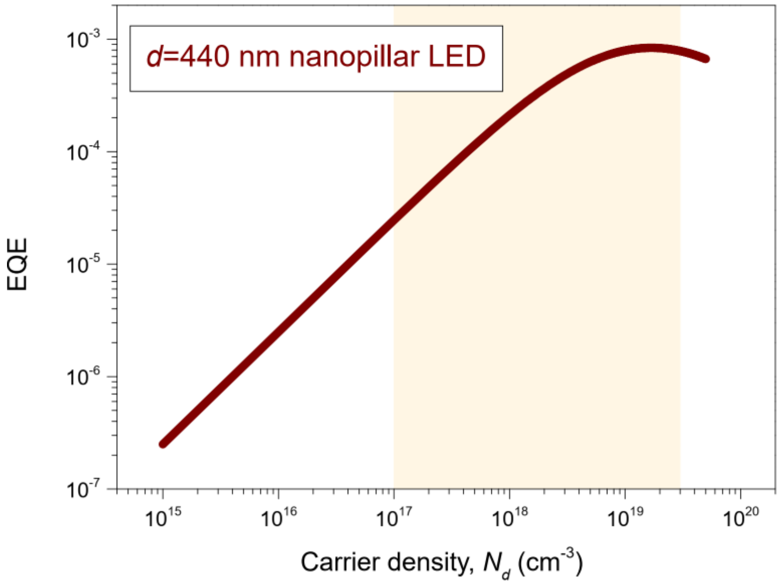


***Figure S12:*** *Estimated external quantum efficiency (EQE) for d=440 nm nanopillar LED.*

**References**


[1] B. Jacob, F. Camarneiro, J. Borme, O. Bondarchuk, J. B. Nieder, and B. Romeira, "Surface Passivation of III–V GaAs Nanopillars by Low-Frequency Plasma Deposition of Silicon Nitride for Active Nanophotonic Devices," *ACS Appl. Electron. Mater.*, vol. 4, no. 7, pp. 3399–3410, Mar. 2022.

[2] G. E. Ponchak and E. M. Tentzeris, "Finite ground coplanar waveguide (FGC) low loss, low coupling 90-degree crossover junctions," *IEEE Trans. Adv. Packag.*, vol. 25, no. 3, pp. 385–392, Aug. 2002.

[3] G. Ghione and C. Naldi, "Analytical formulas for coplanar lines in hybrid and monolithic MICs," *Electron. Lett.*, vol. 20, no. 4, pp. 179–181, Feb. 1984.

[4] B. Romeira, J. Borme, H. Fonseca, J. Gaspar, and J. B. Nieder, "Efficient light extraction in subwavelength GaAs/AlGaAs nanopillars for nanoscale light-emitting devices," *Opt. Express*, vol. 28, no. 22, p. 32302, Oct. 2020.

[5] M. Takiguchi *et al.*, "Direct modulation of a single InP/InAs nanowire light-emitting diode," *Appl. Phys. Lett.*, vol. 112, no. 25, p. 251106, Jun. 2018.

[6] V. Dolores-Calzadilla *et al.*, "Waveguide-coupled nanopillar metal-cavity light-emitting diodes on silicon," *Nat. Commun. 2017 81*, vol. 8, no. 1, pp. 1–8, Feb. 2017.

[7] G. Shambat *et al.*, "Ultrafast direct modulation of a single-mode photonic crystal nanocavity light-emitting diode," *Nat. Commun.*, vol. 2, p. 539, Nov. 2011.
